# Blunt cardiac injury presenting as a left-sided coronary artery dissection

**DOI:** 10.1093/jscr/rjac008

**Published:** 2022-02-11

**Authors:** Alex Park, Daniel R Principe

**Affiliations:** Department of Surgery, University of Illinois at Chicago, Chicago, IL, USA; Department of Surgery, University of Illinois at Chicago, Chicago, IL, USA; Medical Scientist Training Program, University of Illinois College of Medicine, Chicago, IL, USA

## Abstract

The presentation of blunt cardiac injuries (BCIs) following thoracic trauma is extremely varied, classically affecting the right-sided chambers of the heart. In extremely rare cases, BCIs can affect the coronary arteries. Diagnosing a traumatic coronary dissection can be challenging, as not only is presentation highly variable, but dissections are often masked by concomitant injuries. Here, we present the unusual case of a patient presenting to the emergency department following blunt thoracic trauma from an automobile accident. He demonstrated diffuse S and T wave segment elevations on electrocardiogram, and coronary angiography was significant for occlusion of the apical left anterior descending artery and stenosis of the second obtuse marginal artery. The patient was diagnosed with a BCI causing a left-sided coronary artery dissection. This serves as an important reminder that BCIs can manifest in any part of the cardiac anatomy, and should be considered in any patient with a history of thoracic trauma.

## INTRODUCTION

Blunt cardiac injuries (BCIs) describe a wide spectrum of traumatic injuries to anterior chest wall, leading to contusion of the myocardium. Accordingly, the presentation of BCIs is extremely varied, ranging from clinically silent to lethal myocardial wall ruptures [1]. The diagnosis of a BCI can be challenging, particularly given the lack of consensus regarding definition or a gold standard for laboratory testing. On autopsy, the most common finding related to BCI is myocardial contusion followed by chamber rupture, predominantly affecting the right-sided chambers given their anterior positioning in the chest [2]. BCIs affecting smaller cardiac structures are more rare, with select case reports describing injuries affecting the septum, coronary arteries and heart valves. Here, we describe the unique case of a patient presenting to the emergency department following chest trauma due to a motor vehicle accident. The patient was ultimately diagnosed with a BCI, though rather than affect conventional structures, his injury manifested as a left-sided coronary artery dissection. Hence, this is an extremely rare case of left-sided coronary artery dissection as the presenting symptom of a BCI, offering additional insight into a vague and poorly understood clinical entity.

## CASE PRESENTATION

**Figure 1:**
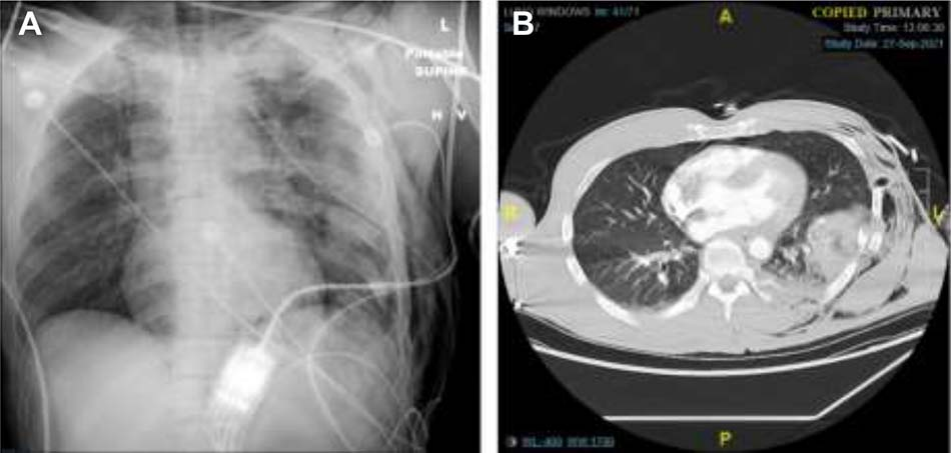
Chest X-Ray and CT imaging showing rib fracture. (**A**) Chest X-ray and (**B**) CT showing fractures to the left ribs.

A 47-year-old man was brought to the emergency department by ambulance following a head-on collision with a tree. The patient was the restrained driver, airbags were deployed, and he required a prolonged 30-minute extrication from the vehicle. En route to the hospital, the patient underwent needle decompression of the left chest due to absent breath sounds, and received 1 unit of erythrocytes. He arrived hemodynamically stable, and was immediately sent for imaging. Chest X-ray and computed tomography (CT) revealed a left hemopneumothorax, as well as a left chest subcutaneous emphysema extending to the left abdomen. We also observed trace left pneumomediastinum, left lung contusions, as well as fractures to left ribs 1–12, thoracic and lumbar transverse processes, a cervical articular facet fracture, and a trace left subdural hematoma (Fig. 1). After placing a left chest tube, the patient was admitted to the surgical intensive care unit (ICU) for traumatic brain injury monitoring.

**Figure 2:**
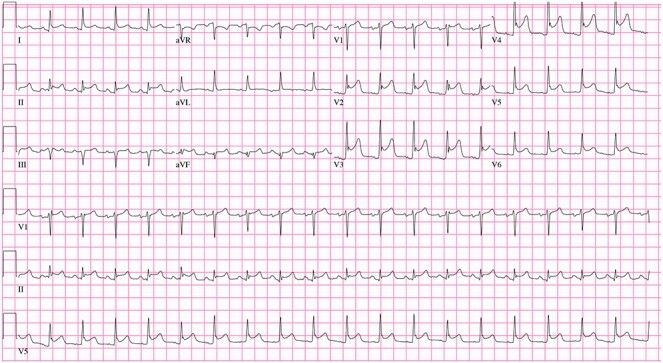
Initial ECG showing ST Elevation anterolateral injury pattern.

**Figure 3:**
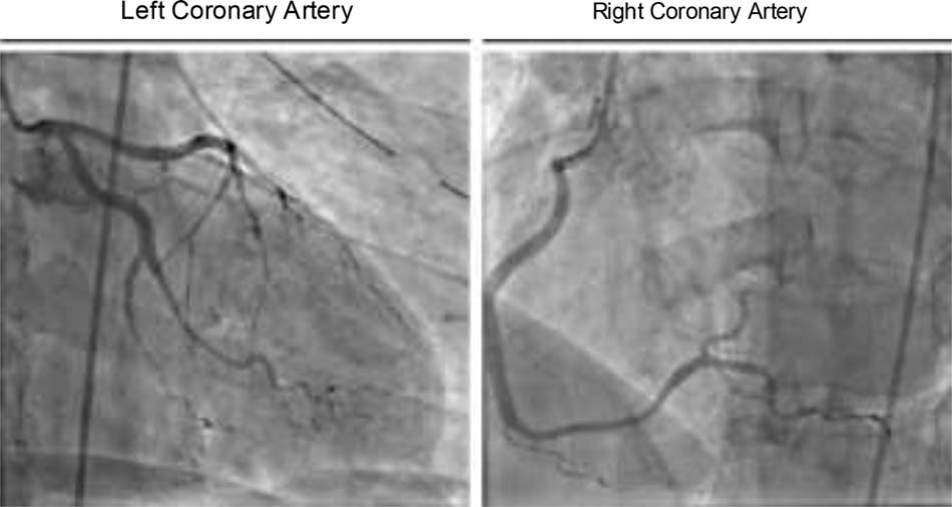
Coronary angiography showing occlusion of the apical left anterior descending artery and stenosis of the second obtuse marginal artery.

In the ICU, the patient was tachycardic and began to complain of chest pain. At this time, a 12-lead electrocardiogram (ECG) demonstrated diffuse S and T wave segment elevations in leads I, V2–6 (Fig. 2). Based on these observations and a significant elevation in serum troponin (0.12 ng/L, normal range 0.0–0.4 ng/L), the patient was oxygenated, given aspirin and nitroglycerine, as well as morphine for pain. Cardiology was consulted and performed a bedside transthoracic echocardiogram but was unable to completely visualize due to the extensive subcutaneous emphysema. Urgent coronary angiography demonstrated occlusion of the apical left anterior descending artery and stenosis of the second obtuse marginal artery with angiographic appearance (Fig. 3), consistent with coronary artery dissections.

Given the distal location of the lesions, small caliber of the vessels and the possibility of propagating the dissections with angioplasty, revascularization was not attempted. A subsequent transthoracic angiogram showed no obvious major reduction in ejection fraction or wall motion abnormalities, although similarly limited to subcostal views like the prior bedside TTE due to the patient’s extensive left-sided subcutaneous emphysema. At this time, we elected for a conservative treatment course with a combination of aspirin and antihypertensive medication. After 11 days, cardiac enzymes normalized, as did the ST segment elevation. The patient was discharged at this time, and has experienced no additional complications.

## DISCUSSION

Coronary artery injury is an exceedingly rare complication of blunt thoracic trauma. While traumatic dissection of coronary vessels can occur, presumptively due to a direct impact causing intimal disruption and thrombosis, the overwhelming majority of BCIs affect the right-sided chambers of the heart. Though rare, traumatic coronary dissection can be life threatening, particularly given the difficulty in diagnosis due to the high variability in presentation and high likelihood of concomitant injuries [3]. When reported, traumatic coronary dissections most frequently affect the left anterior descending artery as seen in our patient, largely due to its vulnerable anterior anatomic position on the heart [2]. Dissection of the circumflex or right coronary or arteries is less common, the latter predominantly associated with acceleration/deceleration injuries due to its position around the relatively fixed and anterior right atrium [4].

As discussed, the clinical course of a spontaneous dissection is highly varied, even without additional cardiac injuries. Although some patients are asymptomatic, others can present with acute coronary syndrome or even sudden cardiac death [5]. As dissections can be life threatening, it is important for care providers to be cognizant of their potential in cases of suspected BCI, regardless of whether patients have clinical signs of coronary injury on intake. This is particularly true in light of recent findings suggesting that such diagnoses can be delayed by up to 5 months after injury [6].

Once diagnosed, the recommended treatment for coronary dissection ranges from conservative treatment to operative revascularization. In many cases, medical management with antiplatelet therapy and blood pressure control is sufficient, with many patients experiencing successful resolution without subsequent cardiac events [7, 8]. Additionally, thrombolytics may be contraindicated for patients with concomitant injuries causing hemorrhage, as seen in our patient with an ongoing hemothorax [9]. For more severe cases, more invasive approaches may be necessary. This can include balloon angioplasty, stent placement and coronary bypass grafting [3]. Hence, treatment should be determined on a case-by-case basis, and should be carefully coordinated between medical and surgical care teams.
